# Application value of dual-sequence MRI based nomogram of radiomics and morphologic features in predicting tumor differentiation degree and lymph node metastasis of Oral squamous cell carcinoma

**DOI:** 10.3389/fonc.2025.1588358

**Published:** 2025-07-15

**Authors:** Bozhong Zheng, Baoting Yu, Xuewei Zheng, Xiaolong Qu, Tong Li, Yun Zhang, Jun Ding

**Affiliations:** Department of Radiology, China-Japan Union Hospital of Jilin University, Changchun, China

**Keywords:** OSCC (oral squamous cell carcinoma), MRI, nomogram, radiomics, LNM

## Abstract

**Background:**

Oral squamous cell carcinoma is a highly invasive tumor. The degree of histological differentiation and lymph node metastasis are important factors in the treatment and prognosis of patients. There is a lack of non-invasive and accurate preoperative risk prediction model in the existing clinical work.

**Objective:**

This study sought to develop and validate a combined model including MRI radiomics and morphological analysis to predict lymph node metastasis and degree of tumor differentiation prior to surgical intervention for oral squamous cell carcinoma (OSCC).

**Methods:**

This study retrospectively included 119 patients which were divided into a training cohort (n=83) and a validation cohort (n=36). To predict lymph node metastasis (LNM) and degree of tumor differentiation, both univariate and multivariate analyses were performed to identify significant features and develop morphological prediction models. Radiomics features were extracted from T2-FS and DWI sequences, followed by feature selection and the establishment of Rad-scores using the LASSO method. Two nomograms was constructed by integrating MRI morphological features with radiomics features. The performance of the models was assessed using the AUC and the Delong test. Calibration curves and DCA were employed to further evaluate the models’ practical applicability.

**Results:**

Nine radiomics features were selected to develop the Rad-scores. The morphological features for predicting LNM are depth of invasion and tumor thickness. The morphological features for predicting the degree of tumor differentiation are ADC value and intratumoral necrosis.In the validation cohort, the nomogram for predicting LNM achieved an area under the curve (AUC) of 0.90 (95% CI: 0.84, 0.97), while the nomogram for tumor grade prediction achieved an AUC of 0.87 (95% CI: 0.76, 0.98), demonstrating excellent diagnostic performance. Calibration curve and decision curve further confirmed the accuracy of nomograms prediction.

**Conclusion:**

Nomograms derived from MRI radiomics and morphological characteristics offer a noninvasive and precise method for predicting degree of tumor differentiation and LNM in OSCC preoperatively. The combined model is an accurate risk prediction model with good clinical benefits and prediction accuracy.

## 1 Introduction

Oral cancer ranks as the eighth most prevalent malignancy globally (1), with oral squamous cell carcinoma (OSCC) representing the predominant subtype (2). The extensive lymphatic and vascular networks associated with OSCC, coupled with the absence of an effective barrier to impede tumor dissemination, facilitate early metastasis of tumor cells. Consequently, this contributes to an unfavorable long-term prognosis for affected patients (3).

The degree of tumor differentiation and lymph node metastasis (LNM) are critical factors influencing the prognosis of patients with oral squamous cell carcinoma (OSCC). The histopathological differentiation of tumor cells serves as an indicator of the tumor’s malignancy (4), with a lower degree of differentiation correlating with a higher propensity for metastasis and a poorer prognosis (5). Currently, there is a lack of non-invasive methods for the preoperative grading of primary lesions in OSCC. The most commonly employed technique is fine needle aspiration (FNA) pathology. However, this method is invasive, and when tumors are located in deeper tissues, the extended puncture distance may facilitate cancer cell implantation and dissemination along the needle tract. Furthermore, studies have indicated that FNA may not adequately assess the tumor’s characteristics due to the limited sample size obtained (6).

The lymphatic system serves as the primary pathway for metastasis in oral cancer, with positive lymph node metastasis indicating a poor prognosis for patients (7). Approximately 40% of patients present with lymph node metastasis at the time of initial consultation (8), and such metastasis can decrease the five-year survival rate by 50% (9). The standard treatment protocol for oral squamous cell carcinoma involves surgical intervention which may be complemented by radiotherapy and chemotherapy based on a comprehensive evaluation of the patient’s specific condition (10). Consequently, an accurate preoperative assessment of lymph node status and tumor histopathological grade is essential to devise appropriate management strategies for patients with oral squamous cell carcinoma.

Radiomics facilitates the extraction of imaging features that are beyond the capacity of human visual assessment, employing high-throughput techniques to reflect tumor heterogeneity at the cellular level (11). Concurrently, magnetic resonance imaging (MRI), with its superior soft tissue resolution, offers enhanced precision in evaluating submucosal spread, infiltration into adjacent tissues, and the status of lymph nodes (12, 13). By utilizing quantitative imaging features such as texture, intensity, heterogeneity, and morphological information derived from MRI scans, radiomics provides a noninvasive approach for the preoperative evaluation of oral squamous cell carcinoma (OSCC), enabling a comprehensive analysis of tumor phenotypes.

In the eighth edition of the Staging Manual by the American Joint Committee on Cancer (AJCC), the depth of invasion (DOI) was introduced as a critical determinant due to its strong correlation with lymph node metastasis (14). DOI is defined as the distance from the deepest point of tumor invasion to the hypothetical healthy mucosal line (15), and it is crucial for achieving an adequate cancer-free margin during surgical resection. Moreover, DOI serves as a significant independent prognostic factor influencing lymph node metastasis and survival outcomes in patients with oral cancer (16). Additionally, researchers (17, 18) have indicated that MRI morphological parameters, such as tumor thickness, lingual distance, and focal apparent diffusion coefficient (ADC) values, are also associated with lymph node metastasis and patient prognosis.

Recently, the integration of Rad-score and clinical indicators within radiomics-based models has gained traction in the investigation of various diseases. Compared to standalone clinical or radiomics models, these combined models demonstrate superior predictive capabilities (19, 20). Nevertheless, there remains a paucity of research concerning the assessment of preoperative tumor characteristics and biological behavior in oral squamous cell carcinoma (OSCC). This study aims to identify clinical, morphological, and radiometric indicators that can predict lymph node metastasis (LNM) and tumor grade in OSCC. Furthermore, it seeks to develop and validate a comprehensive model utilizing magnetic resonance imaging (MRI) to enhance the holistic evaluation of tumors.

## 2 Materials and methods

### 2.1 Patients

**Figure 1:**
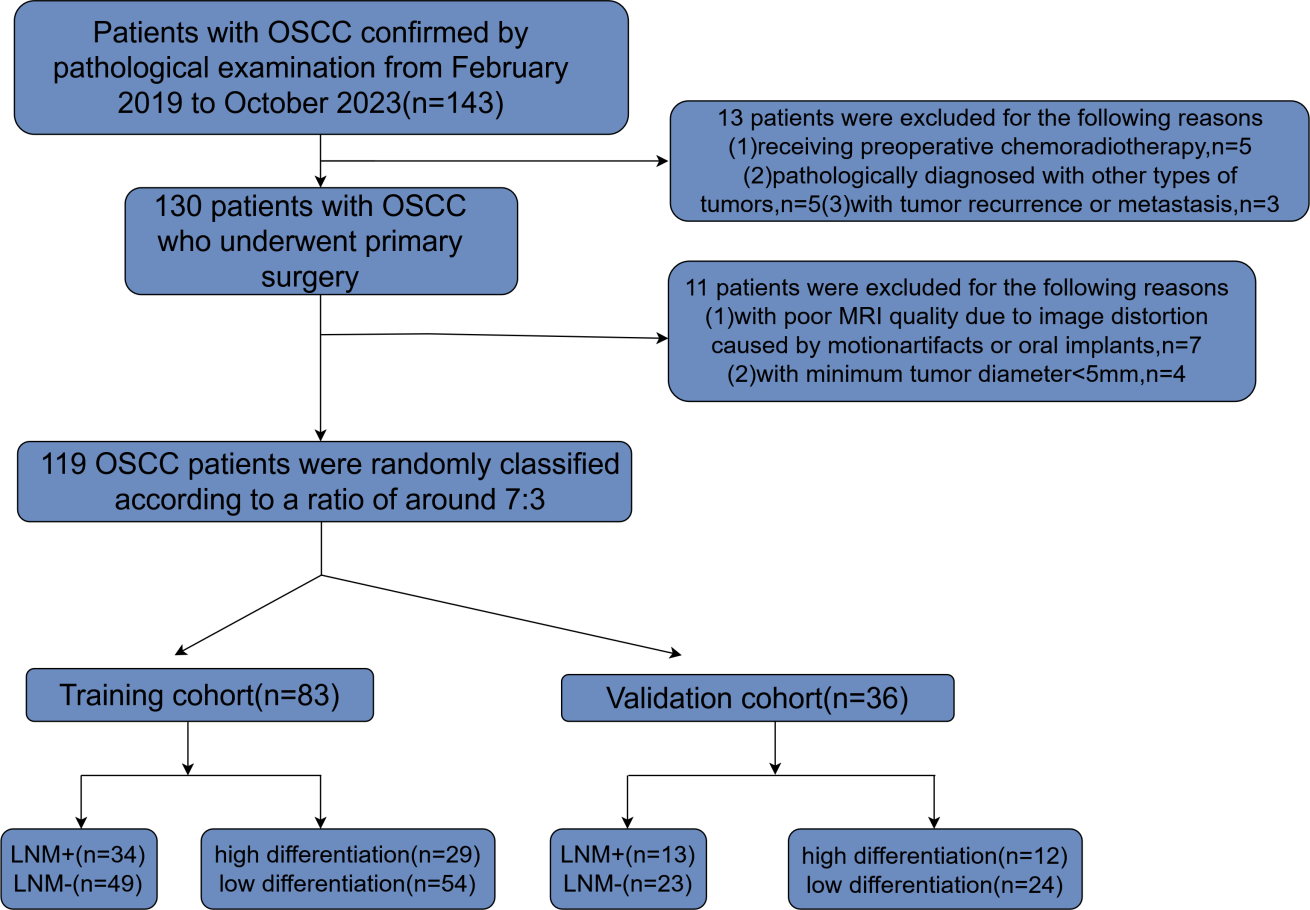
Flowchart depicting the process of patient selection along with inclusion and exclusion criteria. OSCC, oral squamous cell carcinoma; MRI, magnetic resonance imaging; LNM, lymph node metastasis.

From February 1, 2019 to October 1, 2023, a total of 143 OSCC patients undergoing radical tumor resection were admitted to the China-Japan Union Hospital of Jilin University. **Figure 1** illustrates the methodology for the selection and categorization of study subjects. The inclusion criteria are as follows: (1) the primary tumor resection specimens pathology confirmed OSCC and neck dissection (ND) was performed; (2) patients who underwent preoperative magnetic resonance imaging at the China-Japan Union Hospital of Jilin University. The exclusion criteria for patients in this study were as follows: (1) patients undergoing preoperative chemoradiotherapy; (2) patients with pathological diagnoses of other tumor types; (3) patients experiencing tumor recurrence or metastasis; (4) patients with suboptimal MRI quality due to image distortion from motion artifacts or oral implants such as dentures; (5) patients with a minimum tumor diameter of less than 5 mm, rendering them unsuitable for defining the region of interest (ROI). These participants were randomly allocated into training and validation sets at an approximate ratio of 7:3. The median interval from the MRI scan to the complete excision of the tumor was 15 days, with an interquartile range of 8 to 22 days. Postoperative pathological analysis was conducted on all patients to collect tumor and lymph node specimens, facilitating the evaluation of lymph node metastasis and tumor differentiation.

### 2.2 Histopathologic analysis

Pathological examination results were documented in accordance with hospital clinical records. Postoperative pathological sections of oral squamous cell carcinoma (OSCC) were stained using hematoxylin and eosin (H&E). Lymph node metastasis was characterized by the presence of heterogeneous tumor cells within the lymph node tissue. The histological degree of tumor differentiation of OSCC is divided into three categories: well differentiated, moderately differentiated, and poorly differentiated (21). In our study, in order to facilitate grouping while considering that tumors with moderate histological differentiation still have high invasiveness, we referred to samples with tumor histopathology classified as moderately differentiated and poorly differentiated as “low differentiation”, and defined samples with tumor cells classified as highly differentiated as “high differentiation”.

### 2.3 MRI protocols

Magnetic Resonance Imaging (MRI) examinations were conducted utilizing a Siemens Healthineers 1.5 Tesla Avanto scanner, manufactured in Erlangen, Germany, and equipped with an 8-channel phased array neck coil. A relaxation pad was employed to stabilize the patient’s head, ensuring that the shoulder was in contact with the base of the coil. The MRI protocol included axial T1-weighted imaging (T1WI), axial T2-weighted imaging (T2WI), coronal T2WI, as well as axial diffusion-weighted imaging (DWI) and apparent diffusion coefficient (ADC) images. The imaging parameters were as follows: repetition time/echo time (TR/TE) of 5080/87 milliseconds, slice thickness/inter-slice gap of 4.0/0.4 millimeters, comprising 20 slices, with a matrix size of 256 × 256. Finally, we decided to include images of T2FS and DWI sequences for radiomic analysis. The corresponding imaging parameters are shown in **Supplementary Material 1**.

### 2.4 Clinical baseline data and morphological data collecting

The preoperative details comprised 11 elements, which were categorized as follows: (I) standard demographic data of patients, including sex and age; (II) six MRI-based morphological features and three ADC features, which included maximum tumor diameter, tumor thickness, tumor volume, depth of invasion(DOI), intratumor necrosis, tumor margin, focal ADC value(ADC), peripheral normal tissue ADC value (ADC_Norm_), and relative ADC value (rADC). Considering that ADC features can be directly measured in MRI images like morphological features, and for the convenience of grouping, three ADC features will be included in the category of morphological features in the following text. **Table 1** shows the statistics of each variable in the training set. Detailed definitions for each morphological feature are provided in **Table 2**. Two senior radiologists, with 9 and 7 years of experience in neck MRI imaging diagnosis, independently evaluated all MRI morphological features using the PACS system. In cases where the two measurements were similar, the average of both measurements was recorded. In instances of disagreement, a third radiologist (with 11 years of experience in neck MRI imaging diagnosis) intervened, and all three radiologists independently measured the morphological data. The average of the two closest measurements was then recorded.

**Table 1:** Clinical and morphological characteristics in the training cohorts.

Variables		Lymph node status		χ*^2^/t/z*	*p*	The degree of tumor differentiation		χ*^2^/t/z*	*p*
		Negative	Positive			high -differentiation (n=29)	low-differentiation (n=54)		
		(n=49)	(n=34)						
gender	female	11(22.45)	8(23.53)	0.013	0.908	8(27.59)	11(20.37)	0.557	0.456
	male	38(77.55)	26(76.47)			21(72.41)	43(79.63)		
age (year)		58.98±8.30	57.91±10.30	0.522	0.603	58.48±8.85	58.57±9.35	-0.043	0.966
rADC		-0.35(-0.45,-0.27)	-0.37(-0.4,-0.16)	-0.982	0.326	-0.19(-0.34,-0.06)	-0.37(-0.45,-0.21)	-3.751	<0.001
ADC_Norm_		1.33±0.16	1.29±0.13			1.33±0.18	1.31±0.13	0.801	0.426
ADC		0.93±0.24	0.96±0.18	1.227	0.223	1.08±0.21	0.87±0.19	4.744	<0.001
tumor margin	smooth	44(89.80)	23(67.65)	6.328	0.012	24(82.76)	43(79.63)	0.119	0.73
	infiltrative	5(10.20)	11(32.35)			5(17.24)	11(20.37)		
intratumor necrosis	no	40(81.63)	22(64.71)	3.043	0.081	27(93.10)	35(64.81)	7.989	0.005
	yes	9(18.37)	12(35.29)			2(6.90)	19(35.19)		
tumor volume		19.94±12.03	21.29±12.87	-0.49	0.625	19.23±13.59	21.17±11.66	-0.679	0.499
L-max		27.81(20.00,33.36)	32.99(25.03,37.02)	-1.889	0.059	28.21(23.87,35.62)	29.02(23.64,34.18)	-0.043	0.966
Tumor thickness		13.6±6.82	19.1±8.43	-3.283	0.002	15.24±6.26	16.18±8.76	-0.514	0.609
DOI		9.17±3.40	11.8±3.16	-3.568	0.001	10.36±4.02	10.18±3.28	0.223	0.824

Description of the statistical methods involved in **Table 1**: Age, rADC, e.g. as continuity variables: if normal distribution, the standard deviation of all addition and subtraction are represented by standard deviations. The comparison between the two groups of data groups is represented by pairwise independent sample t tests. The continuity data of non-normal distribution is represented by median, 25 quartiles and 75 quartiles. The comparison between the two groups of data groups is represented by Mann-Whitney Test; the comparison between the gender, tumor margin, and intratumor necrosis is represented by chi-square test; the counting data is represented by the number of cases (%). On the two-sided test, a=0.05, that is, p<0.05 has a statistically significant difference.

**Table 2:** Definition of morphological variables.

Morphological terms	Definition
depth of invasion	The distance between the deepest point of tumor tissue infiltration and the theoretical normal basement membrane, regardless of the external part of the tumor(measured in millimeters)
tumor thickness	Taking the maximum tumor diameter as the baseline, the longest distance of tumor infiltration to both sides was measured respectively and the sum of the two was taken(measured in millimeters)
tumor volume	The size of the tumor(measured in cubic millimeters)
L-max	Long wheelbase deviations were measured on the maximum axial images of the lesions (measured in millimeters)
intratumor necrosis	The cystic fluid signal shadow appears inside the tumor, usually presenting as a cystic structure with smooth edges and clearly demarcated from the surrounding solid tumor tissue
tumor margin	If the tumor has blurred edges and does not clearly infiltrate the surrounding normal tissue in MR images, it is considered as “infiltrative”;on the contrary, it is “smooth”.
ADC	A circular area slightly smaller than the lesion was drawn in the area with uniform signal in the largest lesion layer, and three measurements were made and the average value was taken.
ADCNorm	Three ROIs were placed in the normal glands around the lesion, and the ADC value of each ROI was measured and the average value was taken
rADC	rADC=(ADC-ADCNorm)/ADCNorm

### 2.5 Interobserver agreements

The proportion of MRI morphological features identified by two radiologists was assessed for each imaging characteristic, along with the inter-observer agreement. The kappa (κ) value for MRI morphological features indicated a good to excellent level of agreement, ranging from 0.675 to 0.842.

### 2.6 Analysis of clinical and morphological factors

Eleven clinical baseline and morphological characteristics were examined utilizing both univariate and multivariate analytical techniques. The results derived from the training dataset are detailed in **Table 1** and **Table 3**. Factors identified as significant through multivariate analysis (P<0.05) were integrated into the model, facilitating the construction of logistic regression models for predicting lymph node metastasis and tumor grade in oral squamous cell carcinoma (OSCC).

**Table 3:** Multivariable logistic regression analyses for selecting morphological features.

			B	S.E.	Wald*c^2^*	*p*	OR (95%CI)
Lymph node status							
DOI			0.204	0.079	6.634	0.010	1.23 (1.05,1.43)
tumor thickness			0.077	0.037	4.351	0.037	1.08 (1.01,1.16)
tumor margin	infiltrative		0.964	0.668	2.082	0.149	2.62 (0.71,9.70)
	smooth		–	–	–	–	1
constant term			-3.935	1.035	14.444	<0.001	
Degree of tumor differentiation							
rADC			-0.228	2.046	0.012	0.911	0.80 (0.01,43.89)
ADC			-4.773	2.195	4.726	0.030	0.01 (0.00,0.63)
Intra tumor necrosis		yes	1.683	0.829	4.119	0.042	5.38 (1.06,27.34)
		no	–	–	–	–	1
constant term			4.892	2.586	3.578	0.059	

### 2.7 Radiomics analysis

#### 2.7.1 Image segmentation

**Figure 2:**
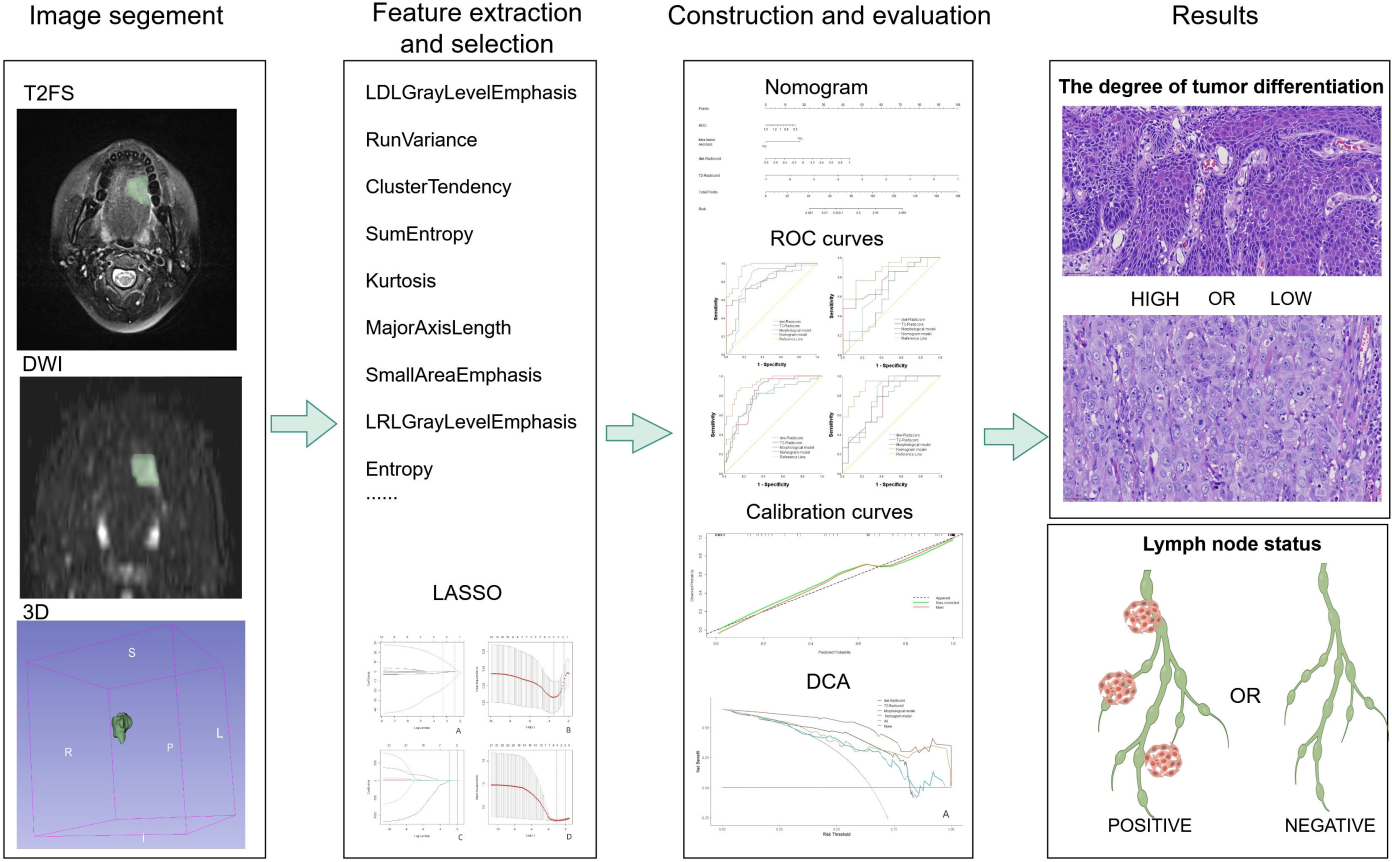
Radiomics analysis flow chart. From left to right, manual segmentation was performed to obtain voxel-based ROIs in 3D slices, radiomics features were extracted using pyradiomics software, features were selected using LASSO regression, and then models were developed, and diagnostic performance was evaluated using ROC analysis at the thickest. The pathological pictures of the results are: above is a light microscopic picture of highly differentiated tongue squamous cell carcinoma, and below is a light microscopic picture of poor-differentiated tongue squamous cell carcinoma. The staining method is HE staining, 40X.

**Figure 2** illustrates the workflow of radiomics. Radiomics features were extracted from the T2-weighted imaging with fat suppression (T2WI-FS) and diffusion-weighted imaging (DWI) sequences with a b-value of 800 s/mm². These sequences were obtained using the 3D-Slicer software (http://www.slicer.org). Radiologist 1 manually delineated the tumor volume of interest (VOI) on a transverse slice to encompass the entire tumor tissue volume, including the cystic necrotic regions, which have been associated with tumor biological behavior in previous studies (22). To assess the repeatability and reliability of the extracted features, a random sample of 30 patients, representing approximately 25% of the total cohort, was selected. The radiologist repeated the segmentation process 45 days after the initial mapping to mitigate recall bias.

#### 2.7.2 Extraction and selection of radiomics features

**Figure 3:**
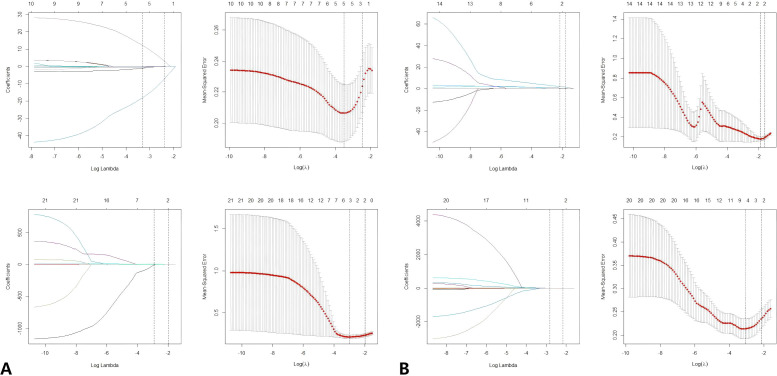
**(A)** LASSO regression was used to select the predictors of T2FS. **(B)** LASSO regression was used to select the predictors of DWI. Radiomics feature selection using LASSO regression algorithm. **(A)** Plot multiple deviations for log (λ). The red dot represents the average deviation value of each model with a given λ, and the vertical line is drawn using the minimum standard at the optimal value, where 12 features have non-zero coefficients. **(B)** LASSO coefficient distribution map of radiomics features. Each colored line represents the coefficient of each feature. LASSO, Minimum absolute shrinkage and selection operator.

The Volume of Interest (VOI) is stored as a label map, and the image is resampled using a trilinear interpolation algorithm. To mitigate the effects of uneven spatial resolution, the new image resolution is standardized to 1x1x1 mm. The processed data were subsequently extracted using the open-source Python package Pyradiomics (version 2.1.0). In total, 851 radiomics features were extracted from both the original and modified images of the T2-weighted imaging (T2WI) and diffusion-weighted imaging (DWI) sequences. These features include 176 first-order statistical features, 11 shape features, 214 features derived from the GrayLevelCo-occurrenceMat-rix(GLCM),157 features from the GrayLevelRunLengthMatrix (GLRLM), 157 features from the GrayLevelSize-ZoneMatrix (GLSZM), and 136 features from the GrayLevelDependenceMatrix (GLDM). Z-score normalization was applied to the eigenvalues from the training set to ensure comparability of features across different dimensions. The mean and standard deviation from the training set were subsequently used to normalize the values in the validation set. Subsequently, a three-step process was implemented for the selection of radiological features. Initially, the Intraclass Correlation Coefficient (ICC) was utilized to assess the consistency of radiomics features. Features exhibiting an ICC of 0.8 or greater were deemed highly repeatable and were retained for further analysis, whereas features demonstrating low repeatability were excluded. In the second step, the correlations between radiomics features within a single sequence (either T2WI-FS or DWI) were assessed using Spearman’s rank correlation analysis. Features with an absolute correlation coefficient (|ρ|) of 0.9 or higher were considered highly correlated. In instances of strong correlation, a single feature was randomly selected for further analysis, while the others were discarded. Finally, the Least Absolute Shrinkage and Selection Operator (LASSO) regression method was applied to the training set to identify optimized features with non-zero coefficients, employing 10-fold cross-validation. **Figure 3** illustrates the screening process for radiomics features using LASSO.

Identify the most predictive radiomics features through a feature selection process, develop a logistic regression model, and calculate the radiomics score (rad-score) using the model’s intercept and coefficients. Subsequently, utilize logistic regression machine learning algorithms to develop comprehensive radiomics models.

### 2.8 Statistical analysis

Statistical analyses were conducted utilizing IBM SPSS Statistics version 25.0, Python version 3.8, and R version 4.2.2. Continuous data following a normal distribution were summarized as the mean ± standard deviation, with comparisons between two groups performed using the independent samples t-test. For continuous data not adhering to a normal distribution, the median along with the interquartile range (25th and 75th percentiles) was reported, and the Mann-Whitney U test was applied for group comparisons. Categorical data were expressed as frequencies and percentages, and group comparisons were conducted using the Chi-square test. In two-tailed tests with a significance level of α=0.05, a p-value of less than 0.05 was considered indicative of a statistically significant difference. The performance of each model was evaluated using an independent test set, with the area under the receiver operating characteristic curve (AUC) serving as the metric for assessment. Calibration and validation of the models were visualized using the “ggplot” package (version 4.1.2) in R, employing the Hosmer-Lemeshow test for goodness-of-fit.

## 3 Results

### 3.1 Statistical analysis of clinical baseline and morphological variables

Ultimately, a cohort of 119 eligible patients (median age: 57 years; age range: 58.98 ± 9.57 years; comprising 91 males and 28 females) was included in our study. **Table 1** presents the clinical baseline data and statistical results of morphological variables of the cases in the training set(n=83). The training set consisted of 83 patients, with 34 positive and 49 negative for lymph node metastasis, and included 29 cases of high differentiation, 37 cases of moderate differentiation, and 17 cases of poor differentiation. The validation set comprised 36 patients, with 13 positive and 23 negative for lymph node metastasis, and included 12 cases of high differentiation, 16 cases of moderate differentiation, and 8 cases of poor differentiation. The results of single factor analysis of included variables are as follows: There were significant differences in depth of invasion (*p* < 0.001), tumor margin(*p*=0.012) and tumor thickness(*p*=0.002) between positive and negative groups. There is significant difference in rADC (*p* < 0.001), ADC (*p* < 0.001), and intra tumor necrosis (*p*=0.005) between high-grade and low-level tumors in histopathology, while there is no significant difference in other clinical and MRI variables.

### 3.2 Establishment of morphological model

A multivariate logistic regression analysis was conducted on variables that showed significant differences in the single factor analysis to identify morphological predictors. The results of multi-factor logistic regression analysis are shown in **Table 3**: In predicting tumor grade, focal ADC and intra tumor necrosis are independent risk factors. In predicting lymph node status, independent risk factors were tumor thickness and depth of invasion. The above variables were used to construct morphological models for predicting tumor grade and lymph node metastasis.

### 3.3 Establishment of Rad-scores and radiomics models

Following the radiomics analyses, the significant predictors of radiological effectiveness identified are: LargeDependenceLowGrayLevelEmphasis,RunVariance,ClusterTendency,SumEntropy,Kurtosis,MajorAxisLength,SmallAreaEmphasis,LongRunLowGrayLevelEmphasis,and Entropy. These selected features are utilized in the computation of the Rad-score, with the corresponding Rad-score formula detailed as follows:

#### 3.3.1 T2FS radiomics features of predicting LNM

The minimum LAM=0.000022, if we take 1 standard error, the coefficient of non-zero mod-el =0.1382144;Rad-score=-2.690716859+LargeDependenceLowGrayLevelEmphasis*0.000534526+RunVariance*1.613346439

#### 3.3.2 T2FS radiomics features of predicting tumor differentiation degree

The minimum LAM =0.000384, if 1 standard error is taken, the coefficient of non-zero model =0.03041584;Rad-score=11.5820663-ClusterTendency*4.1606080-8.4650260*SumEntropy-0.1002329*Kurtosis

#### 3.3.3 DWI radiomics features of predicting LNM

The minimum LAM=0.00017, Rad-score=-1.78041927- MajorAxisLength*0.01596566+ SmallAreaEmphasis* 0.22523289+ LongRunLowGrayLevelEmphasis* 0.10875230

#### 3.3.4 DWI radiomics features of predicting differentiation degree

The minimum LAM =0.000029,if 1 standard error is taken,the coefficient of the model is not zero=0.2011348;Rad-score=-0.8379462+Entropy*0.9363819 + 0.2464287*LongRunLowGrayLe-velEmphasis

Following the acquisition of the Rad-scores, radiomics models, as well as combined models, were developed to predict tumor grade and lymph node metastasis.

### 3.4 Establishment and verification of combined model

**Figure 4:**
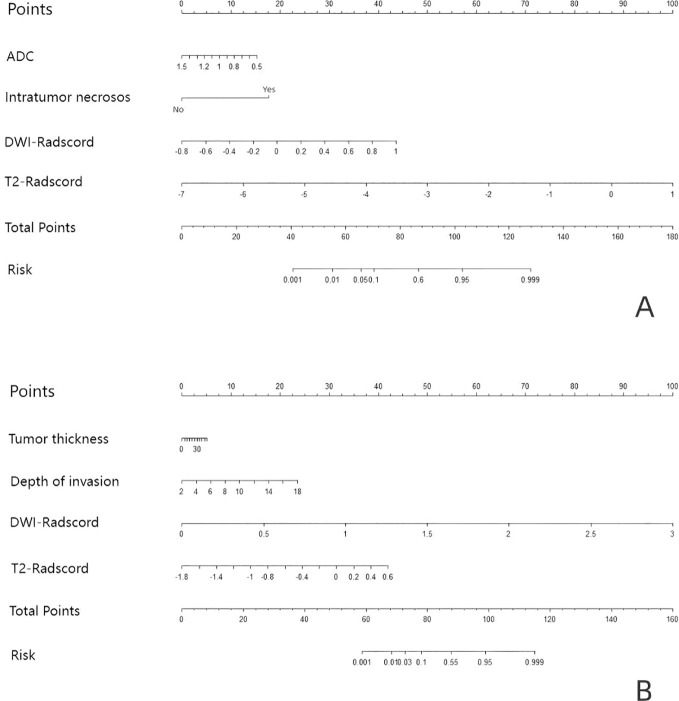
Based on the predictors, nomograms were developed to estimate LNM and tumor grading within the training cohort. **(A)** the nomogram of predicting tumor differentiation degree **(B)** the nomogram of predicting LNM.

**Figure 5:**
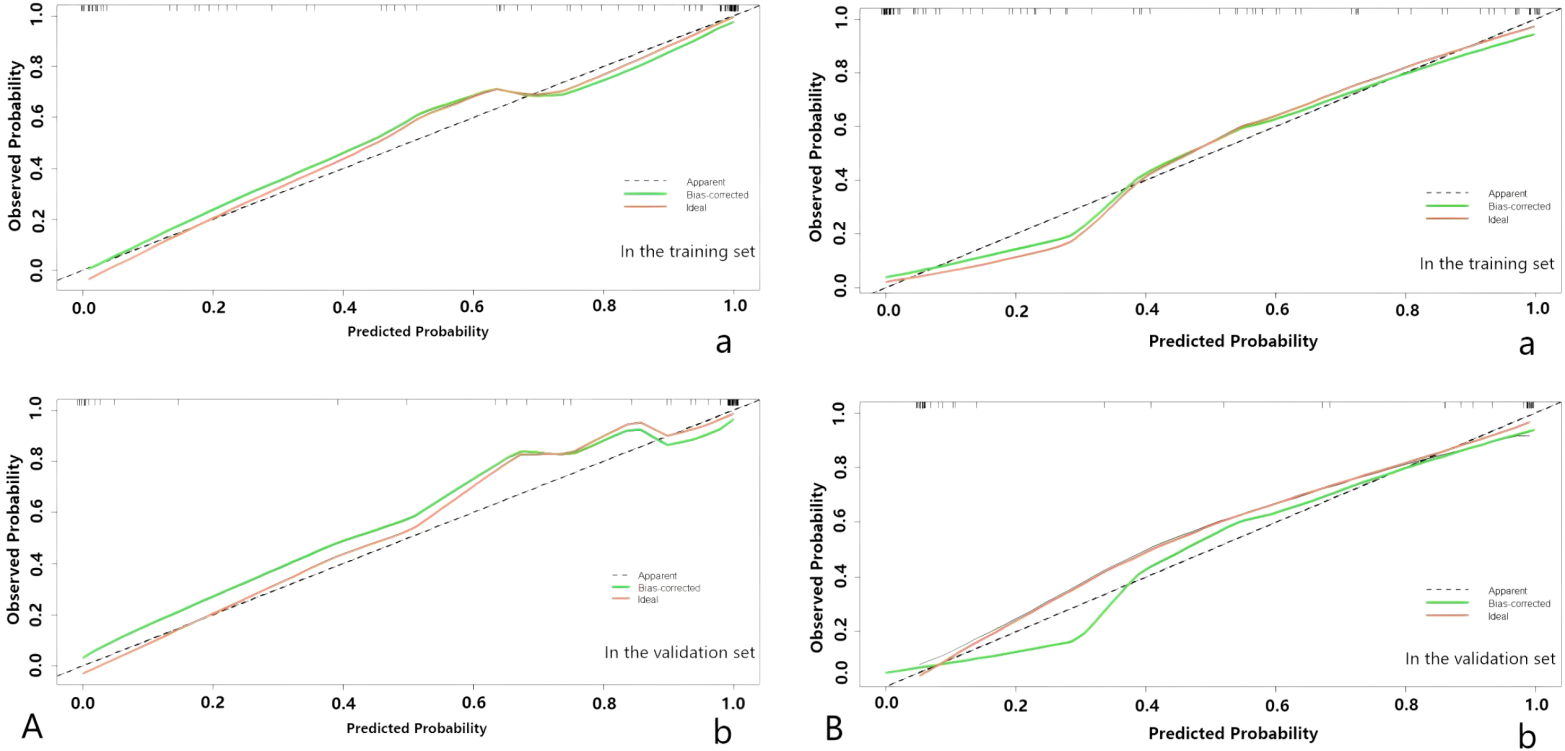
**(A)** The nomogram of predicting tumor differentiation degree. **(B)** The nomogram of predicting LNM. The calibration curve of the prediction model in the training (a) and validation (b) sets; the horizontal axis represents the predicted probability, and the vertical axis represents the actual probability.

**Figure 6:**
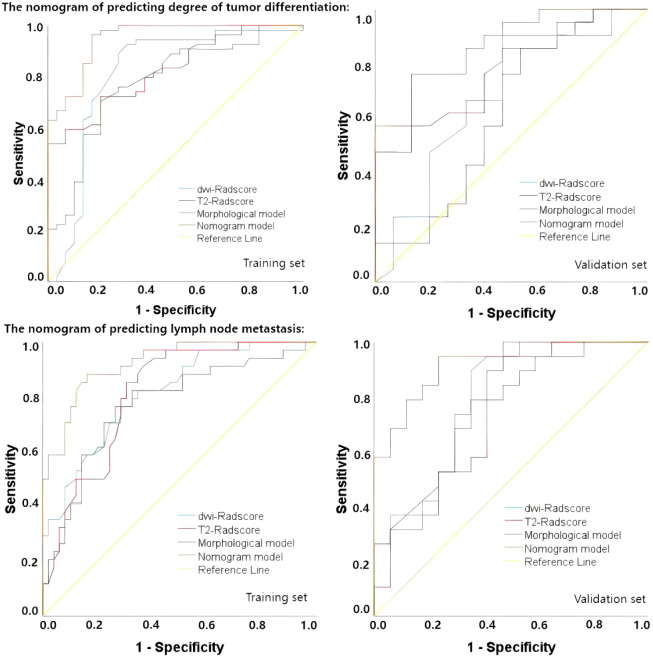
the AUC-ROC curves of predictive nomograms in the training set and the validation set.

**Figure 7:**
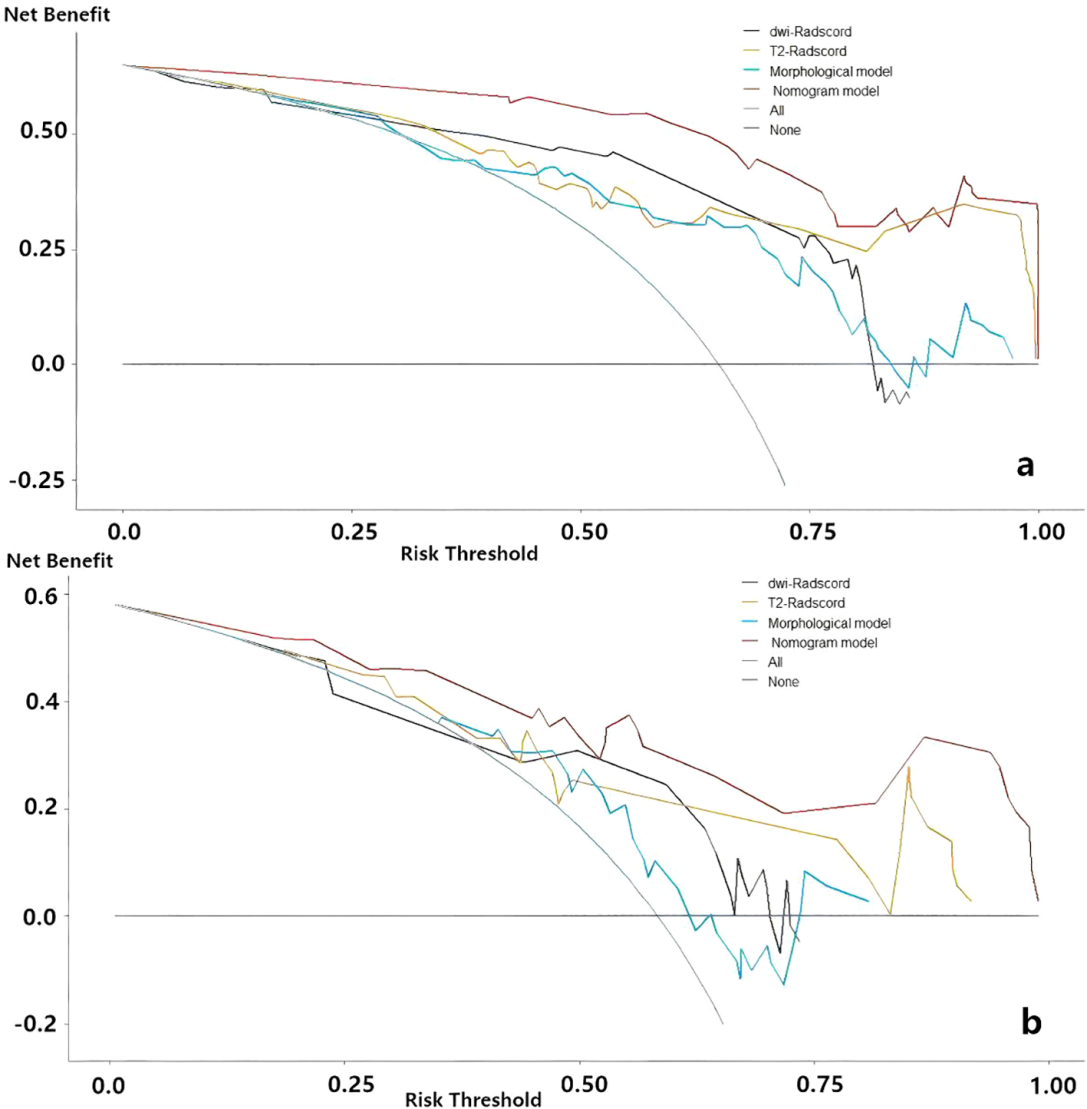
Decision curve analysis for evaluating predictive models. **(a)** the nomogram of predicting LNM **(b)** the nomogram of predicting degree of tumor differentiation.

Upon completing the aforementioned steps, we integrated morphological predictors with Rad-scores to develop two nomogram models using multifactor logistic regression. This model aims to furnish clinicians with personalized quantitative prediction tools (**Figure 4**). Simultaneously, a pairwise comparison was conducted to assess the predictive efficacy among the combined model, the radiomics model, and the morphological model (**Table 4**). In addition to calculating the Area Under the Curve (AUC), calibration and Hosmer-Lemeshow tests were also performed in this study (**Figures 5**, **6**). Ultimately, Decision Curve Analysis (DCA) was employed to evaluate the net benefits across various threshold probabilities, thereby determining the clinical relevance of the nomogram (**Figure 7**).

**Table 4:** Diagnostic performance of different models.

Model		AUC (95%CI)	Sensitivity,%	Specificity,%	PPV,%	NPV,%	Accuracy,%
Tumor grading							
DWI radiomics model	Training cohort	0.81(*0.69-0.93*)	0.944	0.586	0.810	0.850	0.819
	Validation cohort	0.69(*0.50-0.88*)	0.905	0.533	0.731	0.800	0.750
T2FS radiomics model	Training cohort	0.82(*0.74,0.91*)	0.815	0.586	0.786	0.630	0.735
	Validation cohort	0.77(*0.62,0.92*)	0.571	0.800	0.800	0.571	0.667
Morphological model	Training cohort	0.78(*0.67,0.88*)	0.852	0.552	0.780	0.667	0.747
	Validation cohort	0.62(*0.42,0.82*)	0.857	0.457	0.692	0.700	0.694
Nomogram model	Training cohort	0.95(*0.90,0.99*)	0.963	0.793	0.897	0.920	0.904
	Validation cohort	0.87(*0.76,0.98*)	0.762	0.667	0.762	0.667	0.722
Lymph node status							
DWI radiomics model	Training cohort	0.81(*0.72,0.90*)	0.559	0.857	0.731	0.737	0.735
	Validation cohort	0.79(*0.64,0.94*)	0.789	0.647	0.714	0.733	0.720
T2FS radiomics model	Training cohort	0.81(*0.72,0.90*)	0.647	0.735	0.629	0.750	0.699
	Validation cohort	0.74(*0.58,0.91*)	0.789	0.588	0.682	0.714	0.694
Morphological model	Training cohort	0.77(*0.67,0.88*)	0.618	0.796	0.677	0.750	0.723
	Validation cohort	0.74(*0.58,0.91*)	0.684	0.706	0.722	0.667	0.694
Nomogram model	Training cohort	0.92(*0.87,0.98*)	0.765	0.878	0.812	0.843	0.831
	Validation cohort	0.90(*0.84,0.97*)	0.842	0.765	0.800	0.812	0.806

### 3.5 Performance evaluation of the models

The accuracy, sensitivity, specificity, positive predictive value (PPV), and negative predictive value (NPV) of each model are presented in **Table 5**. Regarding tumor grade prediction, the area under the curve (AUC) for the radiomics models (comprising the DWI radiomics model and the T2FS radiomics model) ranged from 0.81 to 0.82 in the training set and from 0.69 to 0.78 in the validation set. In contrast, the morphological model exhibited an AUC of 0.78 in the training set. The combined model demonstrated AUCs *(95% CI: 0.90, 0.99)* in the training set and *0.87 (95% CI: 0.76, 0.98)* in the validation set. The results of the AUC DeLong test, indicate that in the training set, the differences between the combined model and the other models were statistically significant *(p = 0.001-0.005)*. In the verification set, the combined model was significantly different from the T2FS radiomics model *(0.87 vs 0.77, p=0.041)* and Morphological model *(0.87 vs 0.62, p=0.027)* showed statistically significant differences in AUC. In terms of predicting lymph node status, the AUC of the radiomics model was 0.81 in the training set and 0.74-0.79 in the validation set, the AUC of the morphological model was 0.77 in the training set and 0.74 in the validation set, and the AUC of the combined model was 0.92 in the training set and the validation set, respectively (*95 CI: 0.87,0.98*), 0.90(*95 CI: 0.84,0.97*); AUC Delong test among models showed that in the training set, the difference between the combined model and other models was statistically significant (*p=0.001-0.013*). In the verification set, the combined model was significantly different from the T2FS radiomics model (*0.90 vs 0.74, p=0.048*) and Morphological model (*0.90 vs 0.74, p=0.026*) showed statistically significant differences in AUC. ROC curves of each prediction model in the training set and verification set are shown in **Figure 6**.

**Table 5:** Delong test of different models’ AUC.

	Model	Nomogram model	DWI Radiomics model	T2FS Radiomics model
Tumor differentiation degree				
Training cohort	DWI radiomics model	0.005		
	T2FS radiomics model	0.003	0.869	
	Morphological model	0.001	0.636	0.515
Validation cohort	DWI radiomics model	0.093		
	T2FS radiomics model	0.041	0.535	
	Morphological model	0.027	0.639	0.261
Lymph node status				
Training cohort	DWI radiomics model	0.013		
	T2FS radiomics model	0.004	0.976	
	Morphological model	<0.001	0.597	0.523
Validation cohort	DWI radiomics model	0.077		
	T2FS radiomics model	0.048	0.713	
	Morphological model	0.026	0.611	0.990

### 3.6 Clinical benefit analysis of nomogram

Decision curve analysis (DCA) of nomograms used to predict lymph node status and degree of tumor differentiation was performed to demonstrate the practicability of these models in clinical practice by testing them in training sets (23). As shown in **Figure 7**, within a certain threshold range, the use of combined model, radiomics model and clinical model for prediction has a higher benefit than the all-treatment strategy or no-treatment strategy that is more common in clinical work, among which the combined model has the highest benefit.

## 4 Discussion

This study comprehensively investigated the role of MRI-based morphological factors in predicting the degree of tumor differentiation and lymph node metastasis in OSCC, and developed and validated three non-surgical evaluation models. Among them, the combined model combining rad-scores and morphological features has the best effect. In addition, calibration curves showed a high degree of agreement between predicted outcomes and actual outcomes, and decision curve analysis (DCA) showed greater clinical benefit in assessing the risk of these outcomes using a combined model compared to universal treatment or no treatment. Since all the information required in the nomogram can be obtained from preoperative magnetic resonance images, compared with other combined models, this study greatly improves convenience while ensuring diagnostic accuracy. It is believed that in future cancer diagnosis and treatment, this study can provide clinicians with more options for preoperative non-invasive assessment of OSCC.

The degree of tumor differentiation and lymph node status correspond to different treatment methods in patients (24, 25). Kademani et al. (26) even pointed out that tumor histological grading is an independent factor in predicting prognostic survival in patients with OSCC. Each grade reduction in survival rate is about 44%. Compared with highly differentiated tumors, low to medium differentiated tumors were more likely to invade peripheral nerve vessels (27), and the overall survival rate in the low to medium differentiated group was significantly lower than that in the high differentiated group. Therefore, some researchers advocate that in radical OSCC surgery, the resection range of hypodifferentiated tumors should be expanded within the range allowed by head and neck reconstruction surgery (28, 29). Fine needle-aspiration cytology (FNA) is the most commonly used evaluation tool before surgery for head and neck tumors (30, 31). However, in clinical practice, FNA also has defects such as diagnostic effects being affected by operator technology, as well as poor evaluation of overall heterogeneity of tumors due to small sampling volume of tumor tissues (28, 30). The emergence of radiomics analysis provides new ideas for solving this problem.

After 2019, the value of radiomics in predicting the degree of tumor differentiation has been increasingly verified: Yu et al. (32) achieved the prediction of the degree of tumor differentiation of tongue cancer through texture analysis of T2FS images, and the AUC for predicting highly differentiated tumors (G1) in the verification set is as high as 0.81, and the diagnostic performance is good. Ren J et al. (33) performed radiomic analysis of T2 and T1 enhanced images of 80 OSCC patients. In this study, Ren discussed the impact of different classifiers on the diagnostic performance of radiomics models. Due to their small research samples (n=80), they used synthetic minority oversampling technology (SMOTE) for sample expansion, the AUC of LR reached 0.90. In addition to the study of MRI, Li Z et al. (34) also used a nomogram method based on dual energy CT images to predict the degree of tumor differentiation of squamous cell carcinoma (HNSCC) in head and neck. The AUCs of the training set and the validation set are >0.9, further confirming that the joint multifactor model is a reliable tool for predicting tumor information.

Lymph node metastasis is one of the factors that affect the prognosis of head and neck tumors, and is also an important indicator that reflects the degree of tumor spread and invasion (7). In the early stages of the disease, occult lymph node metastasis is a key issue in lymph node management strategies, and its missed diagnosis will cause patients to miss the optimal treatment period and reduce life expectancy (35). When the T phase progressed, a multicenter study (35) found that the regional/distant control rate, tumor-free survival (DFS), and overall survival (OS) of pN0 patients were significantly better than that of the positive group. However, lymph node conditions are difficult to detect through clinical palpation and traditional imaging examinations, and their diagnosis depends on pathological examinations. Imaging omics can extract biomarkers in medical images at high throughput and can effectively predict the status of preoperative lymph nodes (36–40). Romeo V et al. (41) demonstrated that imagingomic analysis based on primary tumor lesions can predict tumor grade and lymph node status of oropharyngeal and oral squamous cell carcinoma based on enhanced CT images. They further discussed the performance of different classifiers, and finally determined that NB, KNN and J48 had good performance: the model predicted tumor grading accuracy to 0.92, and the prediction of lymph node state accuracy to exceed 0.90. However, the sample size of Romeo V et al. is small. This study is small in size (n=40) and more samples are needed to improve feasibility. On the other hand, although the application of various algorithms in prediction was discussed, the optimal model was not determined. In 2022, Wang et al. (42) combined extended pathological information in different peritum ranges (3mm, 5mm, 10mm, 15mm) with radiomics to establish a joint clinical pathological model. The final CR_prim+10_ predicted the best results (AUC = 0.995). In the lymph node-negative subgroup, CR_prim+10_ predicted an AUC of 0.883, indicating that DOI and t2fs-based radiomics predicted OSCC and even occult lymph node status with good performance.

Compared with CT, MRI has the characteristics of multi-sequence imaging, and its combination with radiomics gives greater preoperative prediction potential (43). The patient’s x-ray damage is avoided while achieving accurate predictions. The conventional MRI sequence ensures the availability of basic information such as tumor profile and texture characteristics, while functional sequences such as DWI further supplement other information inside the lesion. For example, tumors with high malignancy are limited by rapid growth rate and dense cell arrangement, thus showing significant differences in DWI compared with low malignant tumors. T2WI can better reflect the detailed characteristics of signal changes, textures and other aspects of different parts of the tumor, and thus perform well in predicting internal heterogeneity of the tumor (44). Tumors of different malignant degrees will show significant differences when undergoing radiomic analysis. For example, the histogram features describe the overall distribution of grayscale in the ROI (45), texture analysis further describes the differences in the distribution of grayscale values ​​in the image (46). Morphological characteristics, first-order characteristics, texture characteristics and higher-level characteristics can fully reflect the differences between different pathological information and achieve prediction results. Our results confirm this: nomograms have excellent results for both lymph node metastasis and tumor differentiation AUCs >0.9.

As people’s understanding of OSCC continues to deepen, researchers have found that morphological factors are important basis for predicting adverse outcomes in patients (18, 47, 48). Moreover, the morphological data obtained based on MRI image measurement has a good numerical correlation with histopathology (49), although its numerical value is slightly larger than the numerical value measured by histopathology, because formalin causes oral tissue to contract (50). The study by Jangir NK et al. (47) suggests that as DOI increases, the probability of lymph node metastasis gradually increases. In our study, the T2FS-based DOI was higher than other histopathological studies and slightly higher than the DOI measured by e-THRIVE in the Jangir NK study because the T2FS sequence is more susceptible to peritum edema, causing the measurement to exceed the actual tumor boundary. Mourad M et al. (35)found that tongue distance and tumor thickness were related to local lymph node metastasis of tongue cancer, but were not related to the ADC value of the lesion itself (p=0.518) or tumor volume size; the results of this study were similar. Morphological factors based on image images have the advantages of being convenient and easy to measure. We hope that more meaningful features will be discovered in future research to further improve the performance of the diagnostic model.

This study has some limitations. Firstly, it is a single-center retrospective study with a relatively small sample size, which poses a risk of overfitting. In future studies, we will include image data from other hospitals to further verify the reliability and generalization of the nomogram. The second is the heterogeneity caused by the location of the lesions: what are the most meaningful morphological features corresponding to each subspecies of OSCCs, which needs further study. In future cohort studies, we will seek to answer this question further if we include sufficient sample sizes of cancers at all four sites. Finally, due to the limitations of our institutional research conditions, we only conducted a binary discussion on pathological differentiation information without conducting more detailed studies. When the relevant research conditions in our campus are complete in the future, we will dig deeper into the pathological information inside the tumor.

## 5 Conclusion

In this study, we proposed a nomogram based on various morphological features and radiomics of magnetic resonance tumor images to noninvasively predict the lymph node metastasis and tumor grade of oral squamous cell carcinoma before surgery. Both nomogram models were predicted to have good diagnostic performance (AUC values of 0.90 and 0.87, respectively, in the validation cohort). The results show that the combined model proposed in this study can help clinicians accurately evaluate the patient’s condition before surgery, select the best surgical strategy, and thus improve the patient’s prognosis.

## Data Availability

The raw data supporting the conclusions of this article will be made available by the authors, without undue reservation.
